# Cross sectional study on prevalence of sickle cell alleles S and C among patients with mild malaria in Ivory Coast

**DOI:** 10.1186/s13104-018-3296-7

**Published:** 2018-04-02

**Authors:** Stephane Koui Tossea, Eric Gbessi Adji, Baba Coulibaly, Berenger Ako Ako, David Ngolo Coulibaly, Philippe Joly, Serge-Brice Assi, Andre Toure, Ronan Jambou

**Affiliations:** 1 Departement de Parasitologie Mycologie, Institut Pasteur de Côte d’Ivoire, BP 490, Abidjan 01, Côte d’Ivoire; 2 Univ Lyon, University Claude Bernard Lyon 1, EA 7424, Lyon, France; 3 Unité de Pathologie Moléculaire du Globule Rouge, Laboratoire de Biochimie et Biologie Moléculaire Grand Est, Hôpital Edouard Herriot, Hospices Civils de Lyon, Lyon, France; 4 Programme National de Lutte Contre le Paludisme, Ministère de la Santé, Abidjan, Côte d’Ivoire; 5 Departement Parasites et Insectes Vecteurs, Institut Pasteur Paris, Paris, France

**Keywords:** Sickle cell anemia, Malaria, Ivory Coast, FRET

## Abstract

**Objectives:**

Sickle cell anemia is due to a mutations on the betaglobin gene, inducing abnormal hemoglobin. In West Africa the main mutations lead to S or C types of hemoglobin. Patients with homozygote mutations seem protected against severe malaria, but not against mild disease. The prevalence of abnormal hemoglobin among patients attending dispensaries for mild malaria is thus unknown. A retrospective study was conducted to update data on the prevalence of S and C hemoglobin among patients attending dispensaries with mild malaria. Enrolment of patients was conducted during in vivo malaria treatment efficacy survey following the 42 days WHO protocol. A group of non-infected pregnant women and a group of patients with fever different from malaria, were also recruited in the same dispensaries.

**Results:**

794 blood samples were included. S and C genotypes were found in all the regions of Ivory Coast with the highest prevalence in the Northern region (S and C genotypes, 27%). In non-infected patients, prevalence of mutations was higher than in malaria patients.

**Conclusion:**

A high proportion of patients with mild malaria carried genetic hemoglobin disorder. This population of high risk must be better investigated to control treatment efficacy and to manage complications.

**Electronic supplementary material:**

The online version of this article (10.1186/s13104-018-3296-7) contains supplementary material, which is available to authorized users.

## Introduction

Nearly 5% of the world population carries mutations in genes of hemoglobin, either as sickle-cell anemia or thalassemia [1]. In the case of sickle cell anemia, the most frequent mutations are on codon 6 of exon 1 of the beta-chain of hemoglobin. In West Africa the main mutations lead to S or C types of hemoglobin. When this glutamin is replaced by valin (6GAG>6GTG) hemoglobin is abnormal and called HbS (Haemoglobin S) and when replaced by lysine (6GAG>6AAG) the abnormal hemoglobin is HbC (Haemoglobin C). Sickle cell anemia refers to an abnormal homozygote genotype (SS or CC), whereas sickle cell trait refers to heterozygote genotype AS or AC inducing mild disease.

The overlapping maps of sickle cell anemia and malaria sustain the hypothesis of the role of malaria in the maintaining of sickle cell trait [2, 3]: i.e. Central Africa with 15% [4], Nigeria 24% [5] and Gabon 28% [6]. However these data are usually outdated as diagnostic facilities are often lacking especially in rural areas as in Ivory Coast [7–10]. Indeed, the proportion of patients with abnormal hemoglobin attending dispensaries for malaria is largely unknown and usually underestimated.

The objective of this retrospective study was therefore to update data on the prevalence of S and C alleles among patients attending dispensary with mild malaria. For this purpose we implemented a FRET (fluorescence resonance energy transfer) molecular method for the detection of mutations on codon 6 of betaglobin gene [11] which allows to re-analyze blood samples collected during drug sensitivity studies conducted over the last 5 years.

## Main text

### Patients, materials and methods

#### Sites of study

Parasitaemia of patients can vary with the malaria transmission level, which correlates with ecological and climatic condition. Sites were as follow.

Abidjan is the economic capital of the country accounting for almost 5 million inhabitants. It is located in the South of the country, with a sub-equatorial climate. Ayamé is located 100 km North–East of Abidjan, in a region of lakes. It is a tropical area where rainfalls occurred almost all over the year and with a very high malaria transmission level. Yamoussoukro is the administrative capital, 248 km North from Abidjan, located in a region of lakes with an equatorial climate. Transmission of malaria is lower with rainfalls accounting nowadays for less than 900 mm/year. Man is the main town of the Western part of the country, 150 km far from Yamoussoukro, surrounded by hills and dense forests. The climate is tropical and transmission of malaria is intense. Bouake is a 1 million inhabitant area, 200 km North from Yamoussoukro, located in a region of savannah. Korhogo is at the frontier with Mali and Burkina Faso. In these two places, the climate is typically sub-Sahelian (dry and hot) and malaria transmission is less intense than in other part of the country.

#### Patient recruitment

The patients included in this study were recruited in six sites during standard in vivo survey of drug efficacy conducted on behalf of the Health Ministry. Protocol of enrolment followed the 42 days WHO (World Health Organization) protocol [12]. Briefly patients attending dispensaries for malaria suspicion were clinically examined and a thin smear of blood was confectioned to confirm malaria according to WHO recommendations [12]. The threshold of parasitemia used for inclusion was set to 2000 trophozoids/μl of blood. They were consecutively enrolled all over the week according to the dispensary procedures. Two supplementary groups of non-infected persons were recruited in the same dispensaries as malaria infected ones to detect potential bias in the prevalence of the malaria patients with abnormal hemoglobin., i.e. pregnant women attending delivery ward (systematic sampling) and patients with fever but without malaria, in Ayamé and Bouake respectively.

#### Biological material

Blood samples were collected according to WHO recommendations [12], either as finger pricked blood dot adsorbed on Whatman^®^ paper or as venous blood.

#### Typing of hemoglobin

Two methods of typing were used according to the samples available. For samples from Yamoussoukro, Ayamé and Korhogo, electrophoresis of hemoglobin was performed on Hydrasys^®^ gel following manufacturer recommendations. In these places blood samples were collected and analyzed in the same time and data were already available in the data bases. For blood samples collected on paper, typing was done retrospectively using FRET approach [11] after DNA extraction and PCR (polymerase chain reaction) amplification. DNA extraction was performed on blood spots using Qiagen^®^ Blood Minikits following manufacturer’s recommendations. For amplification, initial denaturation was carried out at 95 °C for 5 min followed by 32 cycles of denaturation (30 s), hybridization (30 s) and elongation (1 min). Finally, a 5 min elongation was carried out. Hybridization of FRET probes was conducted on RotorGene Q. Denaturation of amplicons was done at 95 °C for 10 min, followed by incubation with probes (Green: LtC640NGCCGTTACTGCCCTGTGGG; Red: CACCTGACTCCTGTGGAGAAGTC_36FAM). For hybridization of probes, the temperature was raised from 45 to 95 °C at a rate of 1 °C/cycle with a stop of 5 s between each step. For each sample the speed of raise of fluorescence was recorded according to the temperature (dfluo/dt curves) (Additional file 1: Figure S1).

#### Statistical analysis

Parasitaemia and age were compared between malaria and non-malaria groups and between regions using Man Whitney (MW) and Kruskal Wallis (KW) tests respectively. Statistical analysis and graphics were done using Statistica 7.0 software. The size of the control groups is calculated to give a 5% confidence interval around a hypothetical prevalence of abnormal hemoglobin of 25%. Thirty-six persons are sufficient and 40 are selected.

### Results

#### Population studied

**Fig. 1:**
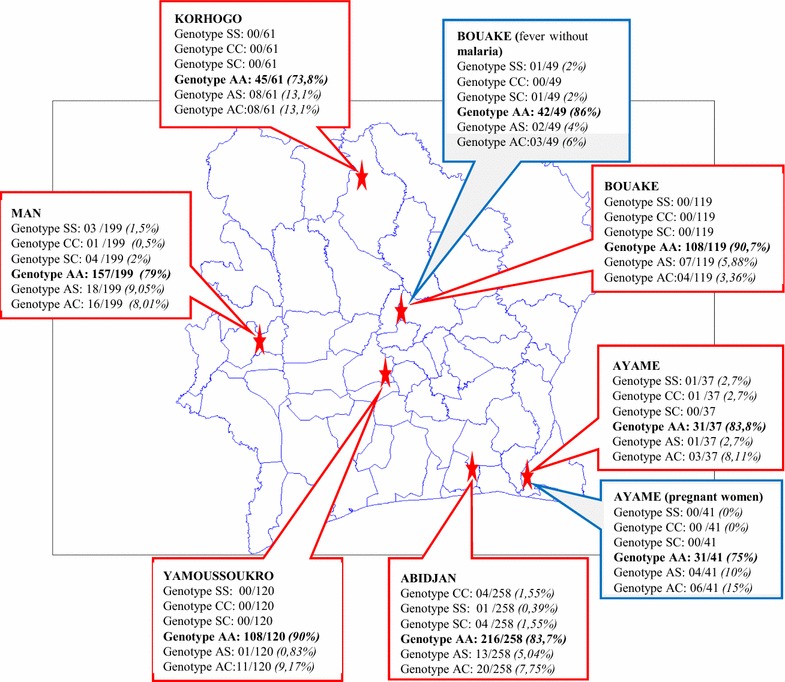
Map of Ivory Coast, with frequency of the genotypes

From patients with malaria 794 samples were genotyped including 422 women (53%) and 372 men (Table 1). Individuals aged from 1 to 10 years accounted for 59% of patients, those between 11–20 years for 23%, and patients over 20 years for 18%. The distribution of persons according to the study sites is reported on the map (Fig. 1). A significant difference of age was observed between regions (KW p < 0.000) with older patients in Abidjan and Ayame. For non-infected groups, 41 pregnant women and 49 outpatients were recruited in Ayame and Bouake respectively.

**Table 1:** Description of patients with malaria and genotypes

AGE (years):	1–4		5–14		15–40		More than 40		Total
Gender:	F	M	F	M	F	M	F	M	
*Abidjan*	*26 (20.5)*	*27*	*36 (29)*	*39*	*15 (11.6)*	*15*	*58 (38.7)*	*42*	*258 (32.5)*
AA	22	25	32	32	13	9	47	36	216 (83.7)
AC	3	1	1	3	1	3	4	4	*20 (7.7)*
AS	1	1	2	1		3	4	1	*13 (5)*
CC			1	1	1		1		*4 (1.5)*
SC				2			2		*4 (1.5)*
SS								1	*1 (0.4)*
*Ayame*					*10 (32.4)*	*2*	*17 (67.5)*	*8*	*37 (4.6)*
AA					8	2	15	6	31 (83.8)
AC					1			2	*3 (8.1)*
AS					1				*1 (2.7)*
CC							1		*1 (2.7)*
SS							1		*1 (2.7)*
*Bouake*	*20 (28.6)*	*14*	*28 (42)*	*22*	*13 (20.2)*	*11*	*8 (9.2)*	*3*	*119 (14.9)*
AA	17	13	26	20	12	10	7	3	108 (90.8)
AC	1		1	1		1			*4 (3.4)*
AS	2	1	1	1	1		1		*7 (5.9)*
*Korhogo*	*10 (29.5)*	*8*	*13 (32.8)*	*7*	*3 (11.5)*	*4*	*7 (26.2)*	*9*	*61 (7.7)*
AA	6	6	10	6	3	3	4	7	45 (73.8)
AC	2	1	1			1	2	1	*8 (13.1)*
AS	2	1	2	1			1	1	*8 (13.1)*
*Man*	*32 (36.2)*	*40*	*22 (22.1)*	*18*	*12 (15.1)*	*18*	*31 (28.5)*	*26*	*199 (25.1)*
AA	28	33	18	15	9	14	22	18	157 (78.9)
AC	1	2		2	2	1	5	3	*16 (8.1)*
AS	3	3	2	1		2	3	4	*18 (9.1)*
CC					1				*1 (0.5)*
SC			1			1	1	1	*4 (2)*
SS		2	1						*3 (1.5)*
*Yamoussoukro*	*17 (28.3)*	*17*	*19 (34.2)*	*22*	*9 (17.5)*	*12*	*16 (20)*	*8*	*120 (15.1)*
AA	15	16	19	19	7	10	14	8	108 (90)
AC	2	1		3	1	2	2		*11 (9.2)*
AS					1				*1 (0.8)*
Total	105	106	118	108	62	62	137	96	794

(), percent

#### Typing of hemoglobin

A total of 545 (68%) samples with malaria were genotyped using electrophoresis and 249 (32%) by molecular method (FRET). All the 90 samples without malaria were genotyped using FRET. Out of 545 samples studied by electrophoresis 483 AA, 24 AS, 34 AC, 1 SS, 2 SC and 1 CC were determined. Out of 249 samples analyzed with FRET, 182 AA, 28 AC, 24 AS, 5 CC, 6 SC and 4 SS (Table 1) were identified. For the non-malaria groups 73 AA, 6 AS, 9 AC, 1 SS, 1 SC were found but no CC. Overall the AA genotype was found in 84% of the patients with malaria whereas genotypes AS plus AC accounted for 14% of the patients, and genotypes SS, SC and CC were present in 2% of the individuals. Among non-malaria persons, AA genotype was found in 81% of the samples with 85% in Bouake and 75.6% in Ayamé (significant difference). However the recruitment of these two groups is not strictly in accordance with the malaria one, and no comparison was done for age and gender.

**Fig. 2:**
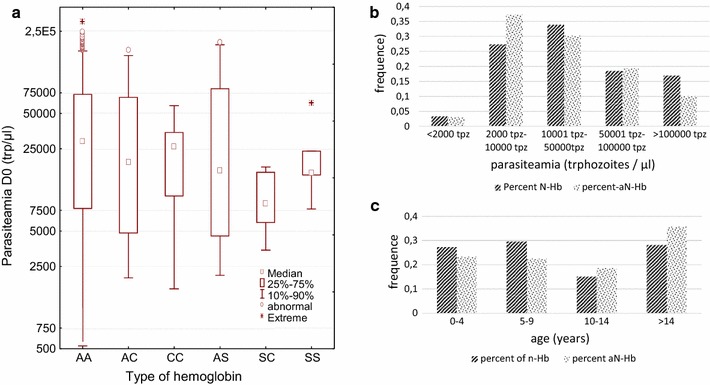
Relation between age and parasitaemia according to the type of hemoglobin. Six genotypes were identified during the studies. Parasitaemia were defined as the number of parasites per microliter as counted on Giemsa stained thin smears [12]. **a** Repartition of parasitaemia according to the type of hemoglobin; no significant difference was observed. **b** Percent of people harboring a level of parasitaemia, according to the normal (Nhb) or abnormal (aNHb) type of hemoglobin; percent are calculated separately for each group. **c** Percent of people harboring normal (Nhb) or abnormal hemoglobin (aNHb) according to age

When we summarize data from patients with or without abnormal hemoglobin, those with abnormal hemoglobin are slightly older than others (Fig. 2c). For patients with malaria, most of mutations were observed in 1–20 years old patients, while only two mutations were found in patients older than 20 years. According to gender, 80% of the CC genotype was observed in women, whereas the other genotypes were found equally in males and females (Table 1).

### Discussion

This study is a retrospective analysis of samples collected during in vivo survey of drug sensitivity of malaria in Ivory Coast. It gives new insights on prevalence of abnormal hemoglobin among patients attending dispensaries for mild malaria. The data sets cannot be considered as representative of the burden of abnormal hemoglobin in the general population as they were collected among patients with mild malaria. Treatment of patients with abnormal hemoglobin must usually be careful as drug efficacy can decrease. Health workers must be aware about this high frequency to pay more attention to the risk of severe malaria or of lower efficacy of the treatment.

During this study, abnormal genotypes were found in all the regions of Ivory Coast, whatever the gender and the age of the patients. The Northern region of Ivory Coast harbored a higher prevalence of abnormal genotypes, followed by the West area, the South and the Center with respectively 27, 21, 17 and 10% of patients with mutated alleles. In this study, the overall prevalence of AS+AC genotypes in malaria patients was 14%, which is higher than the 5% estimated in the Ivorian population [1]. This distribution could be explained by ethnic origin of the populations. In the non-malaria groups, the prevalence of abnormal traits is even much higher with 25% in Ayamé and 14% in Bouake versus 17 and 9.3% for patients with malaria. Prevalence was higher in females (53%) than in males (47%), which is in accordance with Ya et al. [13] but not with Sangaré et al. [9].

For malaria patients, 81% of individuals had parasitaemia between 2000 and 10,000 tpm of blood. Parasitemia differed between region (KW p < 0.0001, Additional file 2: Figure S2), which summarize both an effect of the level of transmission between the regions and an effect of difference of age in the recruitment. Among the malaria group no difference of parasitaemia was found in relation with the different types of hemoglobin (KW test Fig. 2a). The proportion homozygous SS+CC and SC was 2% (5.4% in Ayame). This confirms previous results obtained by Sekongo et al. with 2.7% [10]. It is also in accordance with results obtained in Ghana where prevalence was 74.7, 14.7, 9.1, 0.9% respectively for AA, AC, AS and SS [14]. Using sequencing of DNA a silent mutation was also detected CAT>CAC (histidine) already described by Costa et al. and called A3 allele [11]. Most of CC genotypes were detected using FRET technique which can be related to a higher sensitivity of this molecular technique than electrophoresis [14].

Individuals with a mutated allele harbored usually a higher tolerant for malaria [3, 15, 16]. In Ghana, multivariate regression analysis showed that children with the AS genotype had 79% lower risk of malaria infection compared to those with the AA genotypes [14, 17]. However this is still debating as in other studies no difference was found between people with and without trait [18–21]. The prevalence of CC genotype is also low in the malaria group. This must be studied in deep in the general population as it could be related to a less severe form of malaria for these patients leading to a lower attendance of dispensary, which seems not true for AC heterozygous [22, 23].

### Conclusion

Sickle-cell anemia and malaria are major concerns in Ivory Coast. This study gave more details on the prevalence and distribution of the mutated alleles in the country especially among patients attending dispensaries with malaria. The Northern region accounted for the highest prevalence of abnormal trait with a high prevalence of AC genotypes. However the West area and the South remain the zones where AS and SS genotypes are the most prevalent. The size of the sampling population seems sufficient to consider these results. Due to this very high prevalence of abnormal trait and its potential interaction with drug, the efficacy of the treatments must be carefully controlled in these patients and more studies must be conducted to adapt the scheme accordingly,

### Limitations

The limited number of subjects in the abnormal Hb groups doesn’t allowed an in deep analysis using multivariate analysis taking into account age, gender and region in the model.

This study focused on mild malaria patients.

## Additional files

**Additional file 1: Figure S1.** Molecular typing of betaglobin allele using FRET technic. Fluorescence release is automatically analyzed according to time and temperature (dF/dT). Different alleles are identified according to the temperature of the peak of fluorescence in reference with standards. Dissociation curves showing: A/ one peak for a homozygous genotype AA with the allele A1 (Tm = 61 °C). The Tm of the allele A is between 60 °C and 64 °C; another allele A2 can be identified with a Tm between 52 and 54 °C. B/ Two major peaks for an heterozygous genotype AS with the alleles A1 (Tm = 61 °C) and S (Tm = 66 °C, between 65 °C and 68 °C). C/ A single peak for homozygous sickle cell genotype SS with the S allele (Tm = 66 °C). D/ Two peaks for a patient with sickle cell genotype SC with the alleles S (Tm = 66 °C) and C (Tm = 58 °C; between 56 °C and 58 °C).

**Additional file 2: Figure S2.** Age and parasitaemia for patients from the different regions of Ivory Coast. Parasitaemia are expressed as the number of trophozoites per microliter of blood, counted on Giema stained thick smears. Patients from Korhogo harbored lower parasitaemia.

## Abbreviations

DNA: desoxyribonucleic acid
FRET: fluorescence resonance energy transfer
Hb: hemoglobin
KW: Kruskal–Wallis
MW: Man Whitney
p: p value
PCR: polymerase chain reaction
pH: hydrogen potential
WHO: World Health Organization
µl: microliter
%: percent
°C: degree celsius
