# Supplementary figures and images for: Cross sectional study on prevalence of sickle cell alleles S and C among patients with mild malaria in Ivory Coast
Source: BMC Res Notes. 2018 Apr 2;11:215. doi: 10.1186/s13104-018-3296-7 (PMC5880027; doi:10.1186/s13104-018-3296-7)

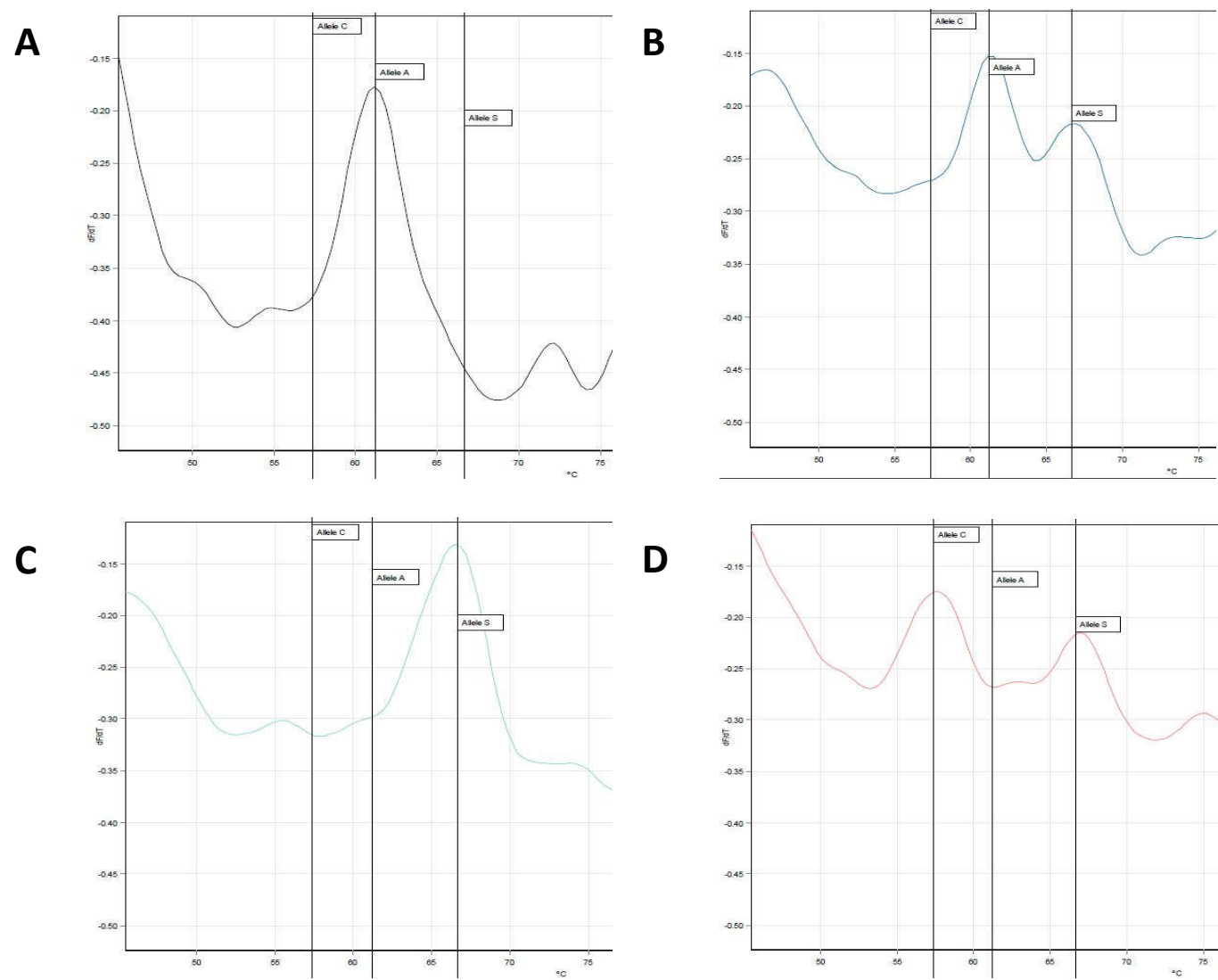

**Figure S1**

Supplement: Supplementary file 1 — Additional file 1: Figure S1. Molecular typing of betaglobin allele using FRET technic. Fluorescence release is automatically analyzed according to time and temperature (dF/dT). Different alleles are identified according to the temperature of the peak of fluorescence in reference with standards. Dissociation curves showing: A/ one peak for a homozygous genotype AA with the allele A1 (Tm = 61 °C). The Tm of the allele A is between 60 °C and 64 °C; another allele A2 can be identified with a Tm between 52 and 54 °C. B/ Two major peaks for an heterozygous genotype AS with the alleles A1 (Tm = 61 °C) and S (Tm = 66 °C, between 65 °C and 68 °C). C/ A single peak for homozygous sickle cell genotype SS with the S allele (Tm = 66 °C). D/ Two peaks for a patient with sickle cell genotype SC with the alleles S (Tm = 66 °C) and C (Tm = 58 °C; between 56 °C and 58 °C). [file 13104_2018_3296_MOESM1_ESM.pdf]

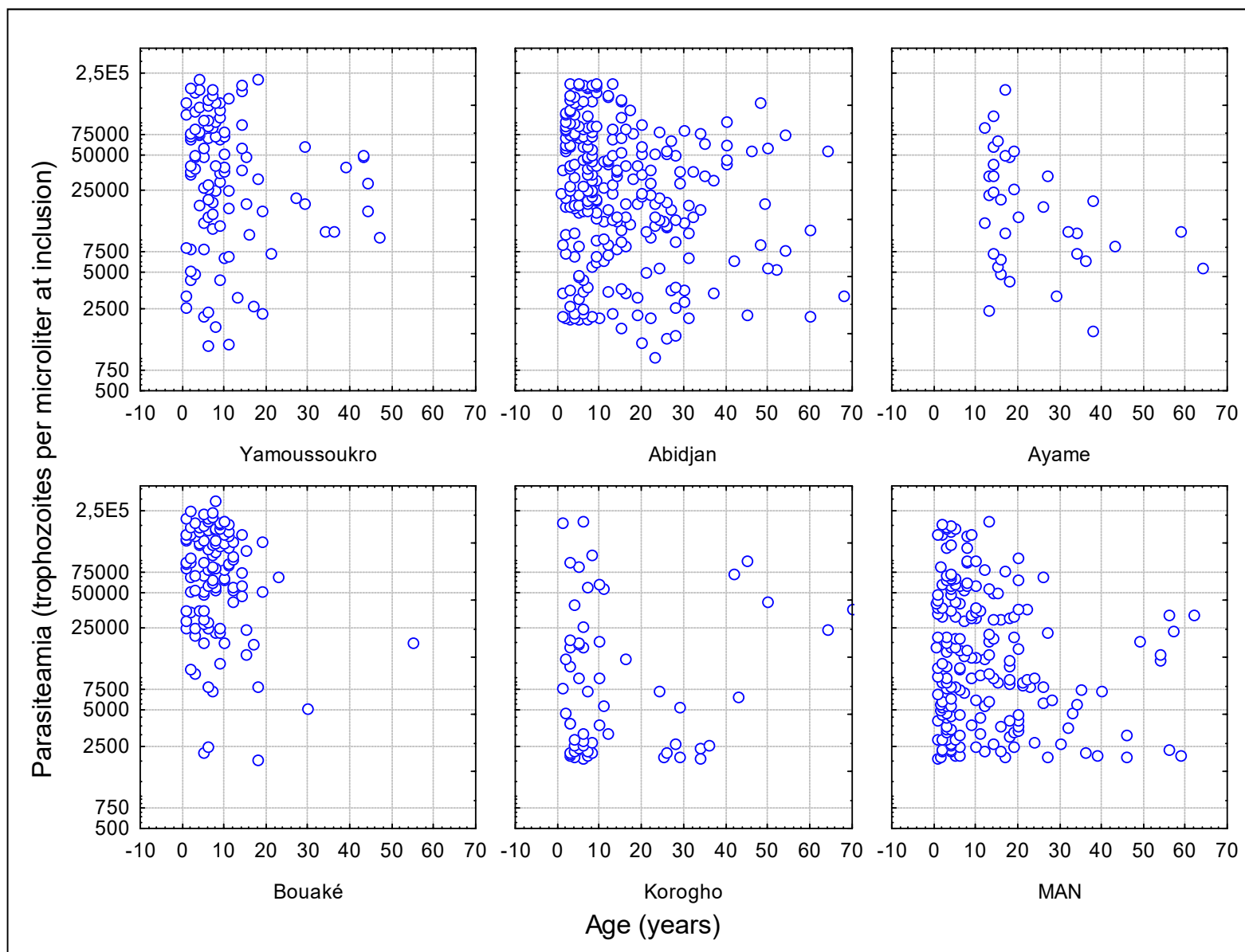

**Figure S2**

Supplement: Supplementary file 2 — Additional file 2: Figure S2. Age and parasitaemia for patients from the different regions of Ivory Coast. Parasitaemia are expressed as the number of trophozoites per microliter of blood, counted on Giema stained thick smears. Patients from Korhogo harbored lower parasitaemia. [file 13104_2018_3296_MOESM2_ESM.pdf]
